# Hyperglycemia and Salivary Gland Dysfunction in the Non-obese Diabetic Mouse: Caveats for Preclinical Studies in Sjögren’s Syndrome

**DOI:** 10.1038/s41598-019-54410-9

**Published:** 2019-11-29

**Authors:** Bujana Allushi, Harini Bagavant, Joanna Papinska, Umesh S. Deshmukh

**Affiliations:** Arthritis and Clinical Immunology Program, Oklahoma Medical Research Foundation, Oklahoma City, OK USA

**Keywords:** Physiology, Autoimmunity

## Abstract

The Non-obese Diabetic (NOD) mouse model for type I diabetes also develops some features of Sjögren’s syndrome (SS). Since the source of the mice and the environment exert a strong influence on diabetes, this study investigated SS development in NOD mice obtained from two vendors. Female NOD mice from The Jackson Laboratory (JAX) and Taconic Biosciences were monitored for blood glucose and pilocarpine-induced salivation. The gut microbiome was analyzed by 16S rRNA sequencing of stool DNA. At euthanasia, serum cytokines and sialoadenitis severity were evaluated. The onset of diabetes was significantly accelerated in JAX mice compared to Taconic mice. Although the gut microbiome between the two groups was distinct, both groups developed sialoadenitis. There was no correlation between the severity of sialoadenitis and reduced saliva production. Instead, salivary gland dysfunction was associated with hyperglycemia and elevation of serum IL1β, IL16, and CXCL13. Our data suggest that inflammatory pathways linked with hyperglycemia are confounding factors for salivary gland dysfunction in female NOD mice, and might not be representative of the mechanisms operative in SS patients. Considering that NOD mice have been used to test numerous experimental therapies for SS, caution needs to be exerted before advancing these therapeutics for human trials.

## Introduction

Reduced fluid secretion by the exocrine salivary and lacrimal glands is a major cause of dry mouth and dry eyes in Sjögren’s syndrome (SS)^1^. Multiple factors like intrinsic glandular defects, innate immune activation, and adaptive autoimmune responses contribute to the development of glandular dysfunction in SS. The Non-obese Diabetic (NOD) mouse model, primarily developed to study type I diabetes, is extensively used to investigate the pathogenic mechanisms in SS^2^. Although under a robust genetic control, environmental factors also significantly affect the development of diabetes in the NOD mouse^3^. The environmental influence is exemplified by studies showing differences in the incidence of hyperglycemia in NOD mice obtained from two different commercial sources, The Jackson Laboratory (JAX) and Taconic Biosciences^4^.

The NOD mice have been used for several preclinical drug studies for SS^5–11^. Since the mice are obtained from different commercial sources or are bred within the investigator’s facility, they are exposed to distinct housing conditions. Thus, it is not surprising to note considerable variations in the incidence, severity, and kinetics of SS in NOD mice. We undertook this study to investigate whether the commercial source of the mice influences the development of SS in NOD mice housed under the same environment.

Our results demonstrate that the NOD mice from JAX had an accelerated onset of diabetes; in contrast, the Taconic mice had a significantly (p = 0.0289) higher severity of salivary gland inflammation. Regardless of the commercial source, mice showed evidence for salivary gland dysfunction, which was strongly associated with hyperglycemia.

## Results

### Salivary gland dysfunction is associated with hyperglycemia

A previous report in the literature showed a higher incidence of diabetes in the NOD/ShiLtJ (JAX) mice compared to NOD/MrkTac (Taconic) mice^4^. Our data also showed that by 20 weeks of age, 80% (12/15) of JAX mice were hyperglycemic with blood glucose >250 mg/dL, compared to 42% (8/19) of Taconic mice (see Supplementary Fig. S1). The 16S rRNA sequencing of stool DNA samples revealed a dramatically distinct gut microbiome composition between the two groups of mice, thereby highlighting the role of gut microbiota in diabetes development in NOD mouse (See Supplementary Fig. S2).

**Figure 1:**
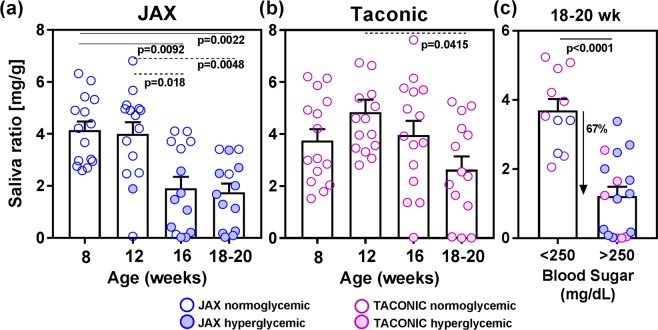
Salivary gland function in NOD mice from JAX and Taconic. Pilocarpine-induced saliva was measured at different time points in female NOD mice purchased from JAX (**a**) and Taconic (**b**). The filled circles represent mice with blood glucose >250 mg/dL. Statistical significance was analyzed by the Kruskal-Wallis test, and the adjusted p values were calculated using Dunn’s multiple comparison test. A p < 0.05 was considered significant. (**c**) The amount of saliva was compared between mice with blood glucose levels less than or greater than 250 mg/dL. Statistical significance was determined by two-tailed unpaired t-test at 95% confidence interval and a p < 0.05 was considered significant.

To evaluate salivary gland function, we measured pilocarpine-induced saliva production at different time points. In JAX mice, there was a significant (p = 0.0092) drop in mean saliva produced at 16 weeks of age (Fig. 1a). In contrast, Taconic mice showed a modest but significant (p = 0.0415) drop at 18–20 weeks of age (Fig. 1b). The analysis of saliva production in JAX and Taconic mice showed that a majority of the mice showing lower saliva amounts had elevated blood glucose. Hence, saliva amounts between hyperglycemic (blood glucose >250 mg/dL) and normoglycemic (blood glucose <250 mg/dL) mice were compared. At 18–20 weeks of age, mice with high blood glucose had significantly lower mean amount of saliva (67%, p < 0.0001) (Fig. 1c).

In our established method of saliva collection^12^, to account for any pilocarpine dosage effects, the amount of saliva produced is normalized by the total body weight of the mouse. This is an important consideration since the mouse body weights are liable to change with age over an extended period of time (see Supplementary Fig. S3). However, the saliva data analyzed without the weight normalization also showed the same results (See Supplementary Fig. S3). Further, regardless of the method used, the functional loss in the hyperglycemic mice was identical (67%, p < 0.0001).

### Salivary gland inflammation does not correlate with hyperglycemia

**Figure 2:**
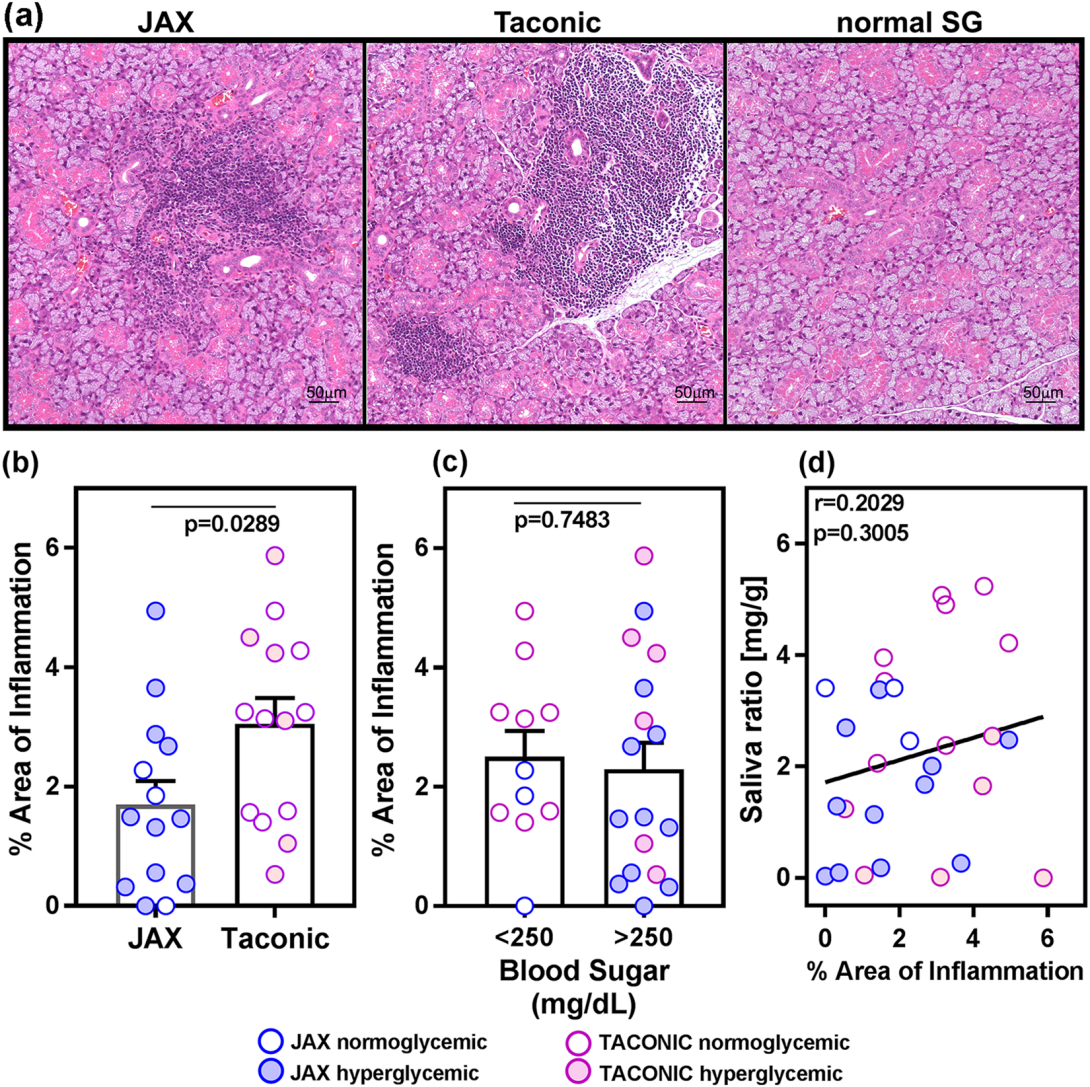
Hyperglycemia does not dictate the extent of sialoadenitis in NOD mice. (**a**) Representative H&E stained sections of submandibular glands from JAX and Taconic NOD mice euthanized at 20–24 weeks of age. For comparison, an image from an area without inflammation is also shown. (**b**) Taconic mice show significantly higher level of inflammation in their submandibular glands, compared to age-matched JAX mice. H&E stained sections of submandibular glands were quantified for the area fraction showing lymphocytic infiltration. Statistical significance was determined by a two-tailed unpaired t-test at 95% confidence level and a p < 0.05 considered significant. (**c**) The % area of inflammation in submandibular glands was compared between hyperglycemic and normoglycemic mice. Statistical significance was calculated by a two-tailed unpaired t-test and a p < 0.05 considered significant at 95% confidence interval. (**d**) Amount of saliva produced does not correlate with the level of inflammation in salivary glands of NOD mice. Pearson correlation test was used for analysis.

The presence of lymphocytic foci in salivary glands is a characteristic feature of SS, which is recapitulated by the NOD mouse model^2^. To accurately quantify the extent of inflammation in the salivary glands, we determined the area fraction of the salivary gland section occupied by lymphocytic infiltrates (Fig. 2a). The submandibular glands from the Taconic mice had significantly (p = 0.0289) higher inflammation than the JAX mice (Fig. 2b). However, the severity of salivary gland inflammation between mice with or without hyperglycemia was not significantly different (Fig. 2c). In addition, regardless of the mouse source, the severity of inflammation in salivary glands did not correlate with salivary gland dysfunction (Fig. 2d). Instead, the mice showed a trend in increased saliva production with higher % area of inflammation. An analysis including only normoglycemic mice (n = 11) showed a similar trend of higher saliva production correlating with increased inflammation. Again, this correlation failed to reach statistical significance (r = 0.48, p = 0.1506).

### Hyperglycemic NOD mice have distinct circulating cytokine profile

**Figure 3:**
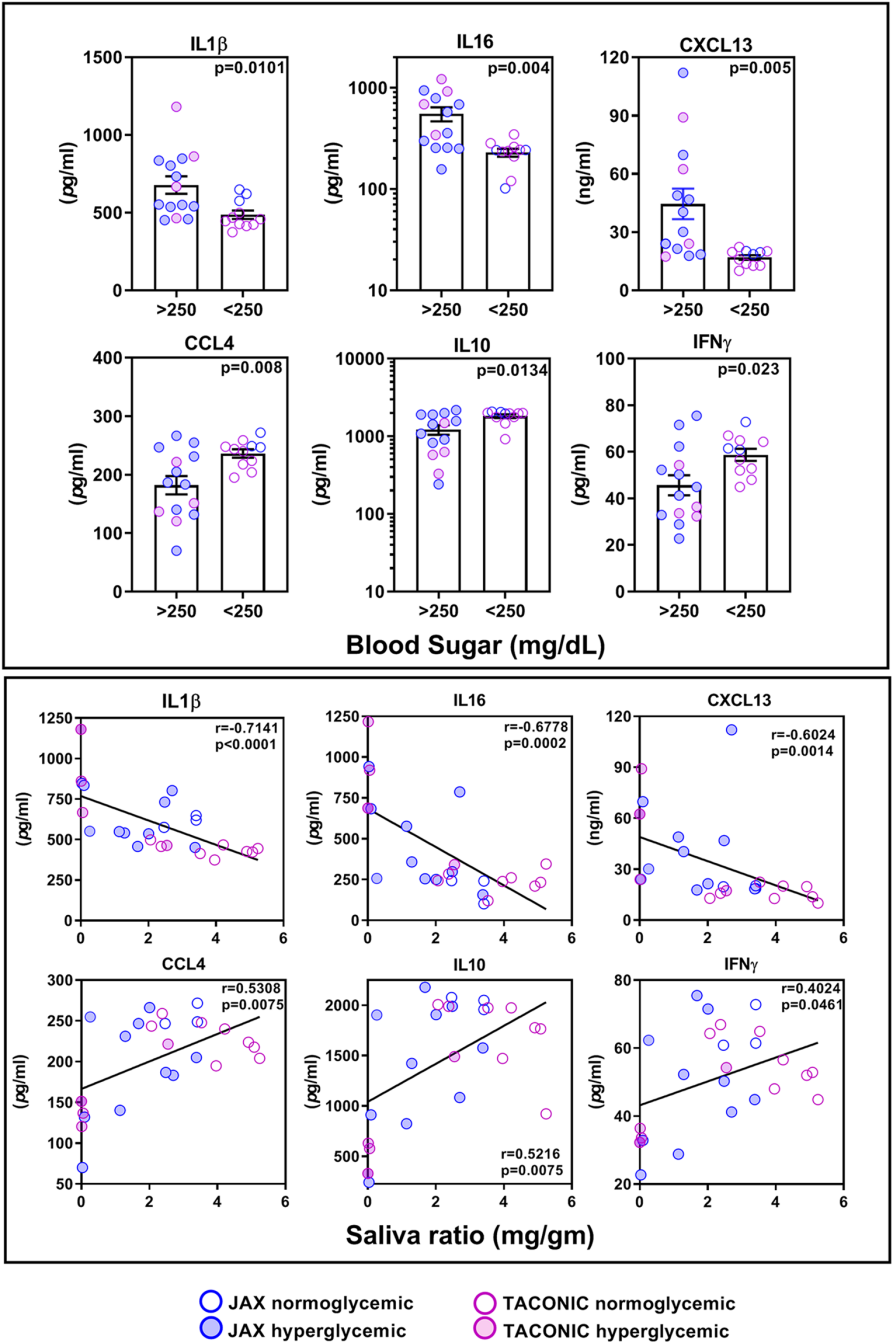
Serum cytokine and chemokine levels in NOD mice and their association with salivary gland dysfunction. (**Top panel**) A 33-plex assay was used to estimate serum cytokine and chemokine levels in NOD mice with blood glucose levels of >250 (JAX (n = 10), Taconic (n = 4), total n = 14) and <250 (JAX (n = 3), Taconic (n = 8), total n = 11) mg/dL. Only cytokines that showed statistical significance (p < 0.05) are shown. Statistical significance was determined using unpaired t-test for all cytokines except CXCL13 and IL10, where a two-tailed Mann Whitney test was performed. (**Bottom Panel**) Correlation between levels of serum cytokines and saliva amount in NOD mice from JAX (n = 13) and Taconic (n = 12). Sera collected at euthanasia (23–24 wks: JAX (n = 11), Taconic (n = 9); 20–21 wks: JAX (n = 2), Taconic (n = 3)) were used for this analysis. To compute correlation, two-tailed Spearman correlation test at 95% confidence interval was used.

To investigate mechanisms responsible for salivary gland dysfunction, a 33-plex assay was used to measure serum cytokine levels in mice with high and low blood glucose (Fig. 3 and see Supplementary Fig. S4). Hyperglycemic mice showed significantly higher levels of IL1β (p = 0.0101), IL16 (p = 0.004), and CXCL13 (p = 0.005), and significantly lower levels of CCL4 (p = 0.008), IL10 (p = 0.0134), and IFNγ (p = 0.023). All three elevated cytokines in the hyperglycemic mice showed a significant inverse correlation between cytokine levels and the amount of saliva produced. In contrast, cytokines elevated in normoglycemic mice showed a modest but statistically significant direct correlation (CCL4: r = 0.5308, p = 0.0075, IL10: r = 0.5216, p = 0.0075 and IFNγ r = 0.4024, p = 0.0461) with saliva production.

### **Differential gene expression in the submandibular glands of hyperglycemic mice**

**Figure 4:**
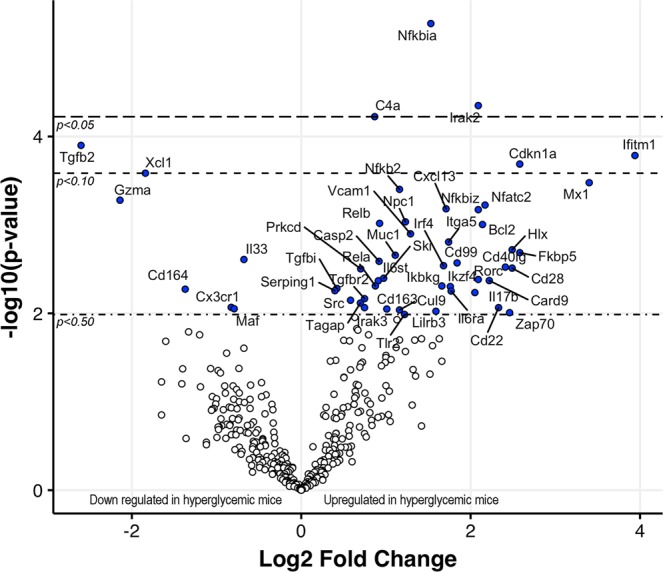
Gene expression analysis in submandibular glands of Taconic mice that were either hyperglycemic (n = 6) or normoglycemic (n = 6). RNA isolated from submandibular glands of mice euthanized at 20–24 wks of age was used for expression analysis. The nCounter mouse Immunology panel (NanoString Technologies, Seattle, WA, USA) was used to analyze the expression of 561 genes. Differential expression analysis was performed by using the nSolver Analysis software (NanoString Technologies, Seattle, WA, USA). Benjamini-Yekutieli False Discovery Rate method was used to calculate the adjusted p values. Please note only 3 genes *Nfkbia*, *Irak2*, and *C4a* showed significant differential expression.

To investigate the effects of hyperglycemia and elevated cytokine levels on salivary gland gene expression, submandibular glands from Taconic mice (six normoglycemic and six hyperglycemic) were used to analyze RNA expression by employing the nCounter mouse Immunology panel (Fig. 4). Although several genes activated downstream of innate immune pathways showed upregulation, only *Nfkbia*, *Irak2* and *C4a* met the cut-off for statistical significance.

## Discussion

Systemic autoimmunity, exocrine gland inflammation, reduced tear, and saliva production are hallmarks of SS. Thus, any therapy that aims to treat SS needs to reverse not only the course of an ongoing autoimmune response but also restore the fluid secretion ability of the exocrine glands. The NOD mice develop autoantibodies, severe inflammation in the submandibular glands, and salivary gland dysfunction and have been widely used in SS research^2^. However, with increasing age, a substantial proportion of female NOD mice develop hyperglycemia limiting the time frame over which SS studies can be performed. The hyperglycemic mice become moribund and have to be removed from the analyses to avoid potentially confounding effects of hyperglycemia on SS phenotype^11^. The consequence is a considerable limitation in the number of mice evaluated and a skewed representation of data from mice retained in the experiment. In this study, using NOD mice from two distinct commercial sources, we show that salivary gland dysfunction is strongly associated with the onset of hyperglycemia and the systemic elevation of pro-inflammatory cytokines. In addition, our study reaffirms the previously reported dissociation between the severity of sialoadenitis and extent of salivary gland dysfunction in NOD mice^13^. Surprisingly, despite dramatic differences in the composition of gut microbiome between JAX and Taconic mice, and distinct kinetics of diabetes, both groups developed sialoadenitis.

The role of hyperglycemia in salivary gland dysfunction is well established. Many individuals with diabetes develop xerostomia^14^. The C57BL/6-*Ins2 Akita*/J (Akita) mice carry a mutation in *Ins2* (insulin 2 gene) and resemble juvenile-onset diabetes mellitus type I^15^. These mice are hyperglycemic without being autoimmune and they do not show any inflammatory cell infiltrates in their salivary glands. Yet they produce little or no pilocarpine-induced saliva, supporting a role for hyperglycemia in salivary gland dysfunction. Although precise mechanisms responsible for hyperglycemia-induced salivary gland dysfunction are not known, the localized production of ROS^16^ and alterations in Ca2^+^ signaling in acinar cells has been proposed to cause functional changes in salivary glands^17^. In our study, serum levels of IL1β showed the most significant negative correlation (r = −0.7141, p < 0.0001) with saliva production. Furthermore, gene expression studies in salivary glands of hyperglycemic mice (Fig. 4) showed significant upregulation in the expression of *Nfkbia* (p = 0.0129) and *Irak2* (p = 0.0487), which are associated with the IL1R signaling pathway.

A previous study in the NOD mice has demonstrated the role of IFNγ in salivary gland disease^18^. NOD mice lacking either IFNγ or its receptor (IFNγR) were protected from autoimmune responses targeting the salivary glands. In our study, the importance of IFNγ in salivary gland dysfunction is highlighted in normoglycemic mice, where serum IFNγ levels showed significant negative correlation with saliva amount (r = −0.7366, p = 0.0097) (see Supplemental Fig. S5). However, IL10, an anti-inflammatory cytokine also showed similar negative correlation with saliva amounts (r = −0.8082, p = 0.0037). It should be noted that even in normoglycemic mice, saliva amount shows significant inverse correlation with blood sugar (r = −0.636, p = 0.035). Collectively these data suggest that direct effects of high blood glucose and cytokines on salivary glands might be the contributing factors for glandular dysfunction in NOD mice. A recent study presented evidence for metabolic disorder in SS^19^. However, whether hyperglycemia contributes to SS pathogenesis can only be evaluated by monitoring SS patients for blood glucose levels.

The NOD mouse has been extensively used to test different therapeutic modalities for SS^5–11^. In many instances, treatments are initiated before the development of hyperglycemia. Whether the beneficial effects on SS phenotype were occurring through the regulation of diabetes has been investigated only in a few studies^10,11^ and remains a critical factor for debate. In this context, a study reporting beneficial effects of resveratrol on SS phenotype suggested that high blood sugar was not a factor for glandular dysfunction in NOD mice^10^. In this report, at 22 weeks, only 2 out of 10 NOD mice had developed hyperglycemia, and these two hyperglycemic mice were among the lowest saliva producers. Of the 8 normoglycemic mice, only one mouse showed lower saliva production. Thus, the conclusion of a lack of correlation between saliva secretion and blood sugar in this report was not supported by robust data.

Considering these caveats in literature and the data presented in this manuscript, the mechanisms for glandular dysfunction in the NOD mouse, might not accurately represent mechanisms operative in SS patients. Thus, preclinical studies employing NOD mice, particularly for restoration of glandular function, need to be rigorously evaluated before any human clinical trials are undertaken. However, it should be noted that the NOD mouse model has provided valuable information on pathways involved in immune cell infiltration in the salivary glands and has utility to investigate mechanisms involved in this process^6,20–22^. Based on our findings, we would like to suggest that the use of genetically modified NOD mice, which show critical characteristics of SS without developing hyperglycemia^23,24^, is a valuable alternative for preclinical studies in SS.

## Methods

### Animals

All animal experiments were approved by the Oklahoma Medical Research Foundation Institutional Animal Care and Use Committee and followed the recommendations set by the National Institutes of Health, USA. Female NOD/ShiLtJ (The Jackson Laboratory, Bar Harbor, ME, USA) and female NOD/MrkTac (Taconic Biosciences Inc., Germantown, NY, USA) were purchased at four weeks of age and housed in barrier cages under specific pathogen-free conditions. The mice were given the feed provided by the respective animal vendors in their facilities. Only female mice were used in this study.

### Blood glucose

Non-fasting blood glucose levels were monitored using the Contour next ONE blood glucose monitoring system (Ascensia Diabetes Care US Inc., Parsippany, NJ, USA) and reading of >250 mg/dL was considered hyperglycemic. Moribund mice were euthanized and tissues were collected.

### Saliva measurement

Pilocarpine induced saliva was measured as described previously^12^.

### Analysis of salivary gland Inflammation

Sections (5 μm) of formalin-fixed and paraffin-embedded glands were stained with hematoxylin and eosin. For quantitation of inflammation, H&E stained slides were scanned on an Aperio CS2 digital pathology scanner and image analysis was performed using the Aperio ImageScope software (Leica Biosystems, Buffalo Grove, IL, USA). An aggregate of >50 mononuclear cells within the salivary gland parenchyma was considered a lymphocytic focus. The total area of the salivary gland and the regions occupied by lymphocytic foci were measured. Since none of the sections showed areas of fatty infiltration, no areas within the salivary glands were excluded from the analysis. The results are expressed as % area of inflammation calculated as: (*Area of inflammation* ÷ *Total area of the section*) × 100. On an average 9.21 ± 0.8 (mean ± SEM) mm^2^ of total salivary gland tissue was studied for each mouse.

### Serum analysis

Sera collected at euthanasia were analyzed for cytokines and chemokines using the 33-Plex Mouse Chemokine Panel (Bio-Rad, Hercules, CA, USA) as described previously^25^.

### Microbiome analysis

Stool samples were collected from the mice at 5–6 weeks of age, and snap-frozen in liquid nitrogen. Stool pellets were shipped to Microbiome Insights (Vancouver, BC, Canada) for 16S rRNA sequencing and analysis. 16Sv4 amplicons generated from stool pellet samples were sequenced on a MiSeq. MiSeq-generated Fastq files were quality-filtered and clustered into 97% similarity operational taxonomic units (OTUs) using the mothur software package [http://www.mothur.org]. The resulting dataset had 9763 OTUs (including those occurring once with a count of 1, or singletons). An average of 43492 quality-filtered reads were generated per sample. High quality reads were classified using Greengenes v. 13_8 as the reference database. The OTUs were aggregated into each taxonomic rank, and the relative abundance of the most abundant ones plotted.

### Statistical analysis

All data were analyzed for Gaussian distribution using the D’Agostino-Pearson omnibus normality test. For analysis of variance between 2 groups, unpaired student’s t-test was used for data fitting Gaussian distribution. For data not fitting Gaussian distribution, a Mann-Whitney test was performed. Both tests were 2 tailed at 95% confidence interval, and a p < 0.05 considered significant. For comparing 3 or more groups either a one-way ANOVA or Kruskal-Wallis test, with Dunn’s multiple comparison was used and a p < 0.05 considered significant. For correlation analysis, either Pearson correlation coefficient (for data fitting normal distribution) or nonparametric Spearman correlation coefficient (for data not fitting normal distribution) was calculated. Both tests were two-tailed, at 95% confidence interval and a p < 0.05 was considered significant. All statistical analyses were performed using the GraphPad Prism software.

## Supplementary information

Supplementary Information

## Data Availability

All data generated or analyzed during this study are included in this published article (and its Supplementary Information Files).
