# Supplementary material for: Hyperglycemia and Salivary Gland Dysfunction in the Non-obese Diabetic Mouse: Caveats for Preclinical Studies in Sjögren’s Syndrome
Source: Sci Rep. 2019 Nov 29;9:17969. doi: 10.1038/s41598-019-54410-9 (PMC6884560; doi:10.1038/s41598-019-54410-9)
Supplement: Supplementary file 1 — Supplementary Information [file 41598_2019_54410_MOESM1_ESM.pdf]

SUPPLEMENTARY INFORMATION.

Hyperglycemia and Salivary Gland Dysfunction in the Non-obese Diabetic Mouse: Caveats for Preclinical Studies in Sjögren’s Syndrome.

Bujana Allushi, Harini Bagavant, Joanna Papinska, Umesh S. Deshmukh.

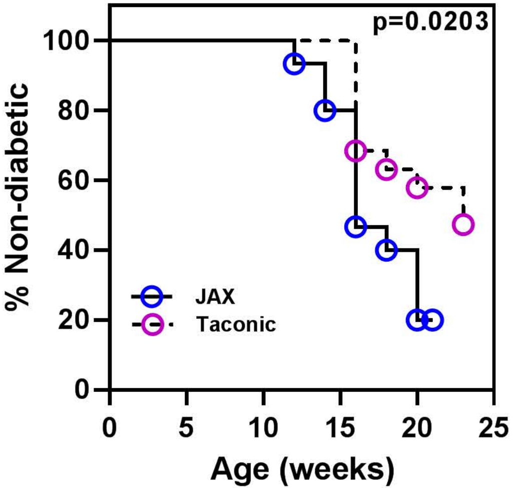

Supplementary Fig S1. Female NOD mice from JAX showed higher incidence and accelerated onset of type I diabetes. Non fasting blood glucose levels were monitored at different time points and mice were considered diabetic if blood glucose levels were >250mg/dL. A total of 15 mice from JAX and 19 mice from Taconic were monitored. Moribund mice were euthanized at different time points. The numbers of mice that became moribund were as follows: JAX mice: week 12 (n=1), week 18 (n=1), week 21 (n=2);
Taconic mice: Week 18 (n=1) and Week 20 (n=4). The difference in survival curves was analyzed by the log-rank (Mantel-Cox) test.

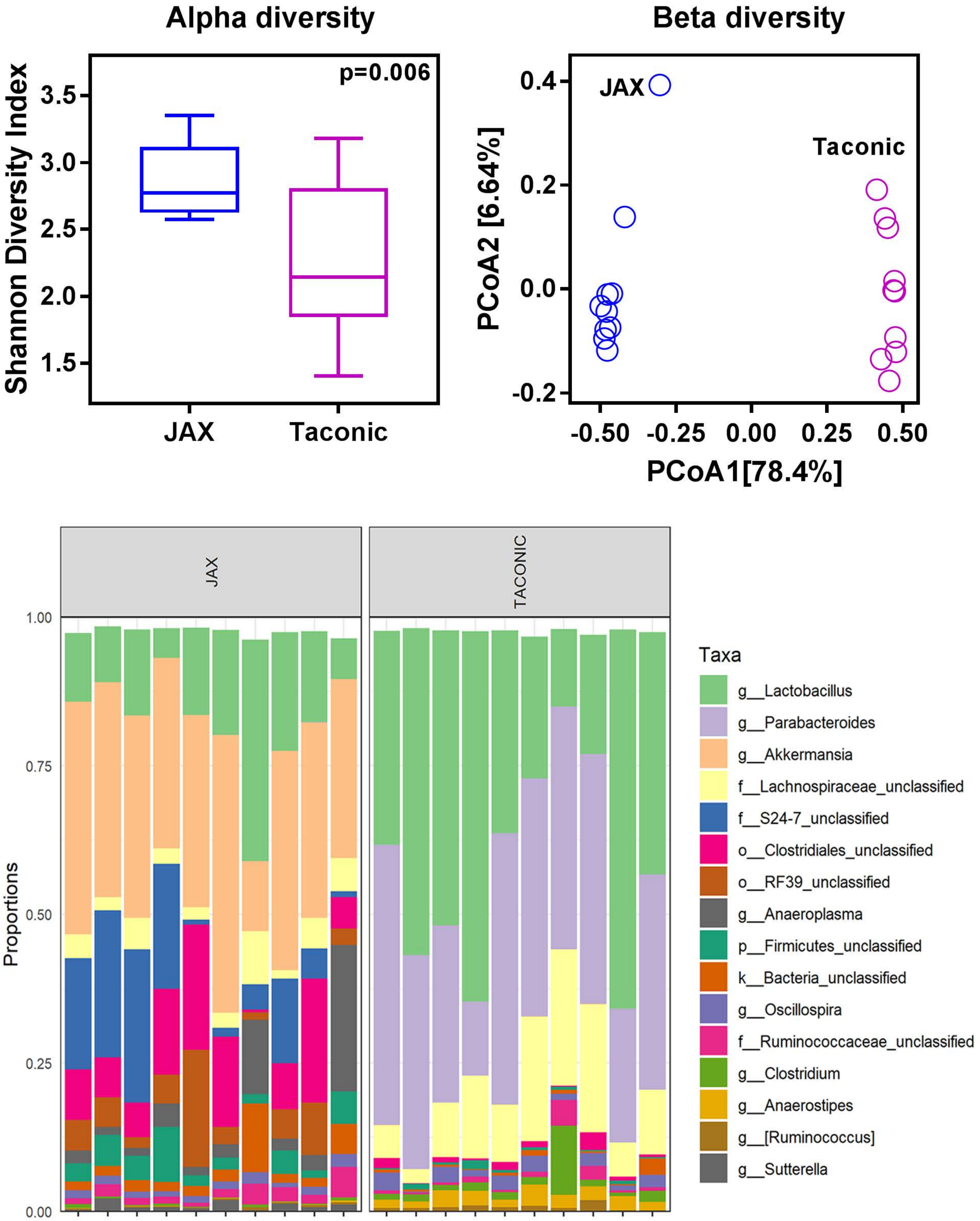

Supplementary Fig. S2. Distinct gut microbiota were seen in NOD mice from JAX and Taconic. Top Panel. The gut microbiome in JAX mice had significantly higher alpha diversity as measured with the Shannon's index (p=0.006). The beta diversity analysis showed significant differences in community profiles according to the source of the mice (PERMANOVA, p<0.001). Differences in the source accounted for 77.8% of the variation of community profiles. **Bottom Panel.** Taxonomic composition at genus level shows that fifteen OTUs were differentially abundant between the two groups. JAX mice were dominated by Akkermansia muciniphila and Lactobacillus spp. These two groups accounted in average for roughly 50% of the community. On the other hand, Taconic mice were dominated by Parabacteroides gordoni and Lactobacillus spp. which accounted for over 75% of the total community. The unfilled portion of the bar plots represent lower-abundance taxa.

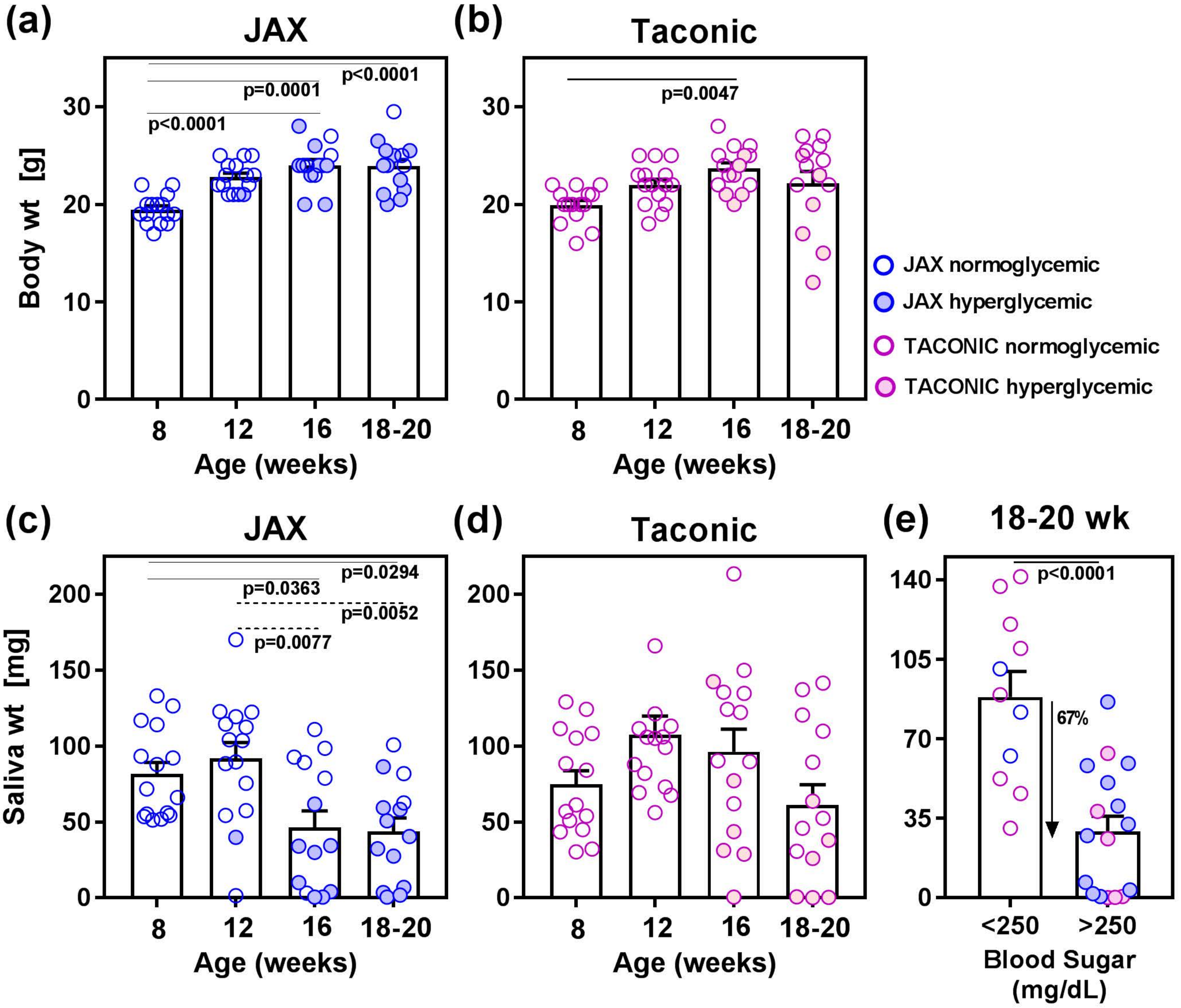

Supplementary Fig. S3. Kinetics of body weight (a & b) and saliva production (c & d) represented as saliva weight in NOD mice from JAX and Taconic. In panels a, b and c one-way ANOVA was used to determine statistical significance. Kruskal-Wallis test with Dunn's multiple comparison was used for analysis of data in panel d. **(e)** Hyperglycemic mice show significant drop (67%, $p<0.0001$) in saliva amount, which is identical to that reported in fig. 1. Unpaired t-test was used to determine statistical significance. All tests were two-tailed with 95% confidence interval and a $p<0.05$ was considered significant.

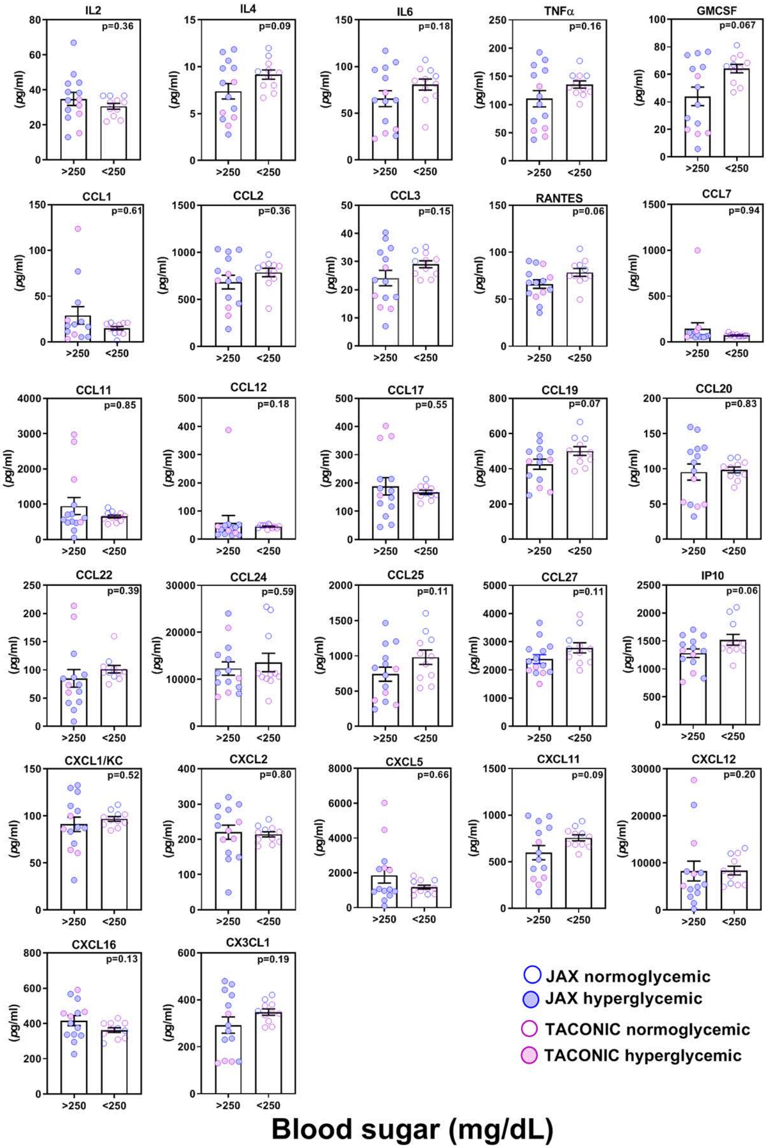

Supplementary Fig. S4. Serum cytokine and chemokine levels in NOD mice and their association with hyperglycemia. A 33-plex assay was used to estimate serum cytokine and chemokine levels in NOD mice with blood glucose levels of >250 (n=14) and <250 (n=11) mg/dL. The 27 analytes shown in this figure did not reach statistical significance ($p<0.05$). Figure 3 in the paper shows analytes that reached statistical significance.

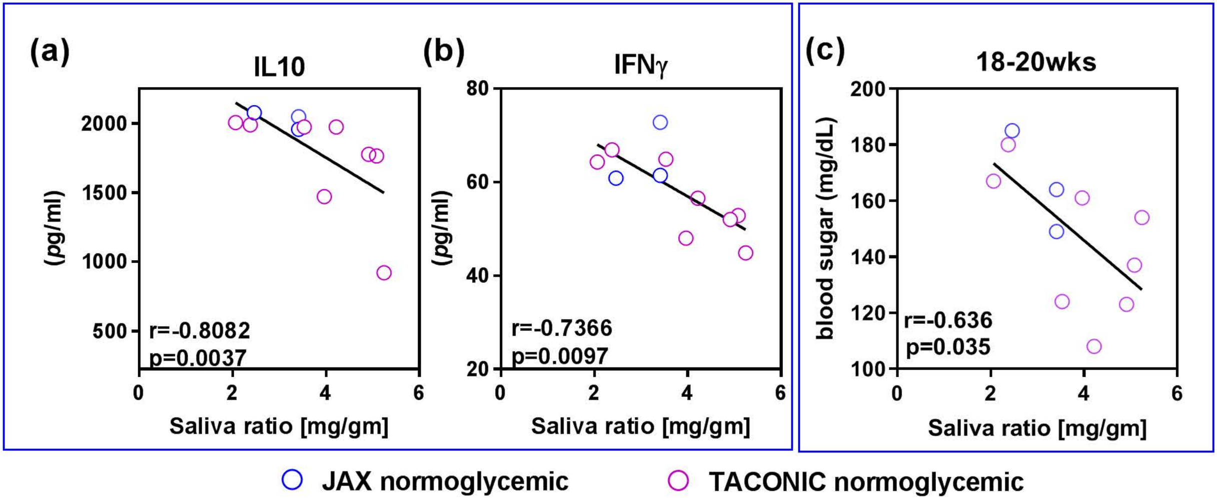

Supplementary Fig. S5. Correlation between cytokine levels and saliva amount (a & b), and correlation between blood sugar and saliva amount (c) in normoglycemic mice from JAX and Taconic. Cytokine levels in sera of mice were measured by multi-plex bead assay. Spearman correlation coefficient was calculated for the analysis of data in panel a, and Pearson correlation coefficient was calculated for the analysis of data in panels b and c. A $p<0.05$ was considered significant.
